# Stakeholder perspectives of Community Mental Health Forums: a qualitative study in Sierra Leone

**DOI:** 10.1186/s13033-020-00382-7

**Published:** 2020-07-10

**Authors:** Ben Adams, Frédérique Vallières, Joshua Abioseh Duncan, Agnes Higgins, Julian Eaton

**Affiliations:** 1 School of Nursing and Midwifery/Trinity Centre for Global Health, Trinity College Dublin and CBM Global, Dublin, Ireland; 2 Trinity Centre for Global Health, Trinity College Dublin, Dublin, Ireland; 3 Mental Health Coalition, Freetown, Sierra Leone; 4 School of Nursing and Midwifery, Trinity College Dublin, Dublin, Ireland; 5 Centre for Global Mental Health, London School of Hygiene and Tropical Medicine and CBM Global, London, UK

**Keywords:** Community Mental Health, Low- and middle-income countries, Traditional leaders, Religious leaders, Sierra Leone, Africa

## Abstract

**Background:**

Mental health is the leading cause of disability worldwide. In the wake of both a civil war and an Ebola outbreak, Sierra Leone ranks as one of the lowest ranked countries on the Human Development Index (UNDP. Human Development Report 2015, Work for Human Development. The United Nations Development Programme; 2015). The WHO identified Sierra Leone among its priority countries for the piloting of its Mental Health Gap Action Programme (mhGAP). Aligned to these efforts, CBM and their affiliated partners employed the use of Community Mental Health Forums (CMHFs), facilitated by Mental Health Nurses (MHNs), as a sensitive and practical way of engaging key community stakeholders to discuss and address issues of mental health. This study sought firstly, to identify factors that affect the successful implementation of CMHFs, as identified by programme participants. Second, the study sought to identify what changes participants perceived as having taken place as a result of their participation in CMHFs.

**Methods:**

10 MHNs and 52 forum participants were purposely selected to take part in key informant interviews and focus group discussions, conducted across eight districts in Sierra Leone. Interview transcripts were analysed across four rounds of coding, using a mixture of deductive and inductive approaches.

**Results:**

Results identified three themes, *Traditional Beliefs and Culture*; *Health System;* and *Inclusive Approaches* as affecting the implementation of CMHFs in their districts. Participants further perceived that their participation in the Community Mental Health Forums resulted in changes taking place across the themes of *Awareness and beliefs*, *Behaviours towards people experiencing psychological distress,* and as leading to greater *Collaboration and cooperation* between formal and informal mental health practitioners.

**Conclusions:**

Results are discussed in the context of the extant literature and a novel framework, that incorporates multiple best practice recommendations and factors which influence the successful implementation of CMHFs is put forward.

## Background

Despite strong evidence of the high global burden of mental health difficulties [1–3], mental health services worldwide remain inadequate and in short supply [4, 5]. Numerous factors contribute to the poor availability and delivery of mental health services, including a lack of political will, poor or weakened health systems, the absence of a specific budget, a shortage of human resources [6], limited knowledge of mental health, and a lack of public awareness [4, 5, 7–9].

On the African continent, where rates of depression are particularly high [10], 70% of countries spend less than 1% of their total health budget on mental healthcare [11] and only 50% of African countries have a mental health policy [4]. In addition, the World Health Organisation (WHO) estimates that 80% of the population in African member states rely on traditional and religious healers for healthcare [12], who often serve as the first point of contact for those seeking support for mental health difficulties [13–16]. This practice may, in part, be due to the lack of availability of health services and weakened health systems [17], but it also is driven by socio-cultural beliefs about the nature and cause of mental health conditions [18].

With a population of over seven million people, Sierra Leone remains one of the lowest ranked countries on the Human Development Index [19] and is still recovering from a 10-year civil war that ended in 2002. More recently, the worst Ebola epidemic in history introduced new sources of psychological distress, including stigma, quarantines and curfews [20], financial stress, economic turmoil and hunger [21]. Traditional practices of caring and laying the dead to rest were not permitted, and interference in the process of loss and mourning amplified the grief and trauma experienced by families and communities across the country [21]. An increase in the prevalence of mental health conditions, including anxiety and post-traumatic stress, was also observed [21]. Existing mental health services is Sierra Leone are minimal and outdated [22]. Despite a great need for mental healthcare and support [18], the country counts only two psychiatrists within its health service, one of which is retired. In addition, there is only one mental health hospital (i.e. The Sierra Leone Psychiatric Hospital) among 22 other government-run general hospitals. Mental health care in Sierra Leone is thus provided by a combination of government, non-government and faith based organisations, as well as traditional and faith based healers. It is reported as many as 90% of those with mental health difficulties in Sierra Leone attend traditional healers for treatment [23]. Consequently, the burden of care for people experiencing psychological distress falls disproportionately on family members, traditional healers, and in more extreme cases, primary health care workers.

Considering these challenges and resource-constraints in mental health programming, there are several recognised strategies that, when implemented, can help support or facilitate the uptake of mental health services. Recognising the importance of traditional, local systems of care during the development of more formal mental health services for example, is recognised as a core element of achieving equitable access to mental health care and support globally [4, 7, 24, 25]. Specifically, ensuring that Western narratives about cause and treatment of mental health difficulties do not dominate local understanding [26] requires a shared and inclusive approach; one that focuses on the sharing of knowledge and ideas about mental health, rather than the globalisation of specific ideas about mental illness and its treatment [27, 28].

To foster a shared and inclusive approach to mental health, programmes should promote the engagement of people experiencing mental health difficulties, their families, and communities, as well as leaders and those in positions of authority, such as religious and traditional leaders and other informal healthcare providers. Evidence suggests that their engagement and participation may increase mental health knowledge and awareness, dispel damaging conceptions surrounding mental health conditions, and change help seeking behaviours [25, 29]. It is also recommended to engage with community members and families to care effectively for people with mental health difficulties as a means to mitigate the shortage of human resources [30] and reduce mistreatment. Finally, it is suggested that strategies to improve pathways to mental healthcare promote collaboration between formal and informal community-based care providers [31]. By creating a synergy between these parallel systems of care [31, 32], various stakeholder groups can work together to improve capacity and increase the available human resources. Despite these recommendations, at the time of writing, few mental health initiatives in LMICs had managed to incorporate all these strategies into a single intervention.

In 2010, the WHO identified Sierra Leone as a priority nation for the piloting of its Mental Health Gap Action Programme (mhGAP) [24, 33]. The ‘Enabling Access to Mental Health in Sierra Leone’ (EAMH-SL) programme, run by CBM, City of Rest, the Community Association for Psychosocial Services and University of Makeni, was developed to strengthen the national mental health system’s ability to respond to existing treatment gaps [22] and lack of psychosocial support. In line with mhGAP, EAMH-SL had three components: (i) build capacity for service delivery at district and primary level, (ii) develop a national mental health coalition to provide advocacy and peer support, and (iii) develop a national mental health awareness and community engagement programme. The latter consisted of various interventions, primarily Community Mental Health Forums (CMHFs), which incorporate the aforementioned best practice recommendations into a single programme, including recognising the importance of traditional and local systems of care, a shared and inclusive approach, community engagement, and fostering collaboration.

### The intervention: Community Mental Health Forums (CMHFs)

In line with best practice recommendations, the CMHFs were designed as a systematic, sensitive, and practical way of meaningfully engaging with members of local communities. The objectives of the CMHFs are listed in Table 1. Involving both formal and informal care providers, including traditional healers and religious leaders, the goal of the forums is to harness practices that promote mental wellness, whilst also preventing practices associated with rights abuse and negative outcomes. Designed to facilitate and encourage open dialogue, active listening, understanding and mutual learning between all of the stakeholders, the CMHFs’ content were developed during the course of numerous multi-stakeholder participatory workshops and involved: exploring local understandings of psychological distress, mental health difficulties and healing; raising awareness through the provision of mental health education in line with mhGAP; education surrounding distress, mental health difficulties, assessment, treatment and available services; and reaching agreement on complementary roles in supporting community members with mental health difficulties, referral procedures and collaborative care.

**Table 1:** Objectives of the Community Mental Health Forums

Engage with communities to share understandings of issues related to mental health
Increase mental health awareness among informal care providers (traditional healers, traditional leaders and religious leaders)
Encourage positive changes in the way people with mental health difficulties are treated within and by their communities
Address negative myths and beliefs surrounding mental health conditions
Reduce the stigma surrounding mental ill health
Strengthen the relationship between trained mental health nurses and the communities where they work
Facilitate collaboration between both formal and informal care providers
Improve access to and utilisation of services by strengthening and defining a referral mechanism

Despite the growing evidence for the importance of engaging programme stakeholders to mitigate the social, human, and economic costs of mental health conditions globally [3], current literature rarely features the perception and experiences of participants as part of the evaluation of such programmes. This study therefore sought to incorporate the experiences of participants to identify the factors that contribute towards the successful delivery of Community Mental Health programming in Sierra Leone. To achieve this goal, the study had two objectives: (i) to identify the factors that stakeholders perceive as affecting the successful implementation of the CMHFs in this context and (ii) to understand the perceived changes as a result of taking part in the CMHFs, if any. Ultimately, this study sought to identify the factors that impact on mental healthcare, mental health interventions, service provision and utilisation within the Sierra Leonean context, towards the improvement of community mental health programmes in this region.

## Methods

### Study participants and procedures

The study used a two-stage purposive sampling approach. First, eight of the 14 districts that had received the CMHF intervention were purposively selected in consultation with the EAMH-SL team. The eight districts were chosen based on (i) an even rural–urban mix, (ii) the availability of the mental health nurses (MHNs) who had facilitated the forums in each district on dates that would allow a logical travel itinerary (reducing cost and time) and (iii) ensuring representation from each geographical province (north, east, south and west). Next, a maximum variation sampling strategy was employed [34] to ensure that a diverse range of people were included and each stakeholder group were represented (Table 2). To be eligible for inclusion in the study, MHNs and attendees had to be over the age of 18 and have attended at least one of the 3 days of the CMHF held in their district. In total 10 MHNs and 52 forum attendees were recruited, from a possible 21 MHNs and 1241 attendees that had participated in the CMHFs, nationally. Data collection took place over a 3-month period, between April and June 2016.

**Table 2:** Summary of the study participants

Forum facilitators	No. male	No. female	Total
Mental health nurses	5	5	10
Forum attendees			
Traditional healers	8	3	11
Imams	9	0	9
Pastors	9	0	9
Traditional birth attendants (TBA)	0	1	1
Community chiefs	5	0	5
Youth leaders	2	3	5
District councillors	3	0	3
Community chairladies	0	5	5
Mammy queens^a^	0	4	4
Total	41	21	62

^a^Historically, the Sierra Leone Peoples Party started the practice of crowning women with the party’s cap and calling them mammy queens due to their political organisation and leadership skills. Now an official title, and although the role has changed somewhat, this practice still continues within communities. Politicians elect mammy queens who take on various roles representing their community and as leaders within their community

### Data collection

Data were collected using one-to-one semi-structured interviews (SSIs) and focus group discussions (FGDs). SSIs were conducted with MHNs, while FGDs were conducted with forum attendees. Interviews took place within the government hospital in each selected district or in the EAMH-SL office in Freetown. The SSI guide contained nine loosely structured, open ended questions, allowing the interviewer to pursue an idea or response in more depth, when appropriate [35]. Given the high level of English spoken by MHNs, all SSIs were conducted in English by the principal investigator (BA) and lasted an average of 50 min. The FGDs lasted an average of 75 min and were conducted by a trained enumerator from Sierra Leone, who was fluent in the most common local languages: Krio, Mende and Temne. The focus group guide consisted of twelve open ended questions and participants were encouraged to explore the issues of importance to them. The SSI guide was piloted with MHNs not involved in the forums and the FGD guide was piloted with stakeholders who had attended a CMHF. Revisions to the interview guides included additional questions and the rephrasing of others to ensure they were not misinterpreted across the different dialects. Responses acquired during the pilot phase were not used during the analysis.

### Data analysis

Focus group discussions were transcribed in their original language by two transcribers with research experience, before being translated into English. The transcribers cross-checked each other’s work to prevent the loss of original meaning through the transcription process. All SSI’s were transcribed verbatim in English to maintain context with the inclusion of non-verbal cues.

Four rounds of data analysis (coding) were conducted using a mixture of deductive and inductive approaches [36, 37]. First, transcripts were manually analysed using pre-existing codes, based on the barriers, facilitators, expected outputs and outcomes, as identified in the study’s theoretical framework. A second round of coding was then used to identify new codes not represented within this initial framework [35], inductive guidelines by Strauss and Corbin [38] were followed. Taken together, this approach allowed for the identification of themes from the literature, while at the same time allowing new themes to emerge from the data. A third phase was then used to generate categories, defined as concepts at a higher level of abstraction representing experiences and perceptions of stakeholders that appeared to be significant elements of the CMHFs. Finally, the data were examined selectively to identify the most commonly reoccurring categories and the importance placed on them by participants. That is, categories that were not directly related to the CMHFs per se were not included (e.g. personal anecdotes unrelated to the topic at hand). The data collection and analysis stages of this research took place concurrently to ensure themes were grounded in the data [39]. Rigour in the analysis was enhanced by comparing the data from two different data sources (SSIs and FGDs). This method of triangulation has previously been described as a validity procedure [40] which encourages reflexive analysis [35], eliminates bias and increases truthfulness [41].

## Results

A total of six categories were identified as a result of the various rounds of coding (see Additional file 1). Three categories were identified as affecting the implementation of mental health interventions, such as CMHFs: *Traditional Beliefs and Culture*, *Health System,* and *Inclusive Approaches*. A further three categories were identified that represent perceived changes as a result of the introduction of CMHFs in Sierra Leone. Specifically, changes were reported to have taken place internally, in the form of changes to *awareness and beliefs*; externally, in the form of changes in *behaviours towards people experiencing psychological distress;* and at the level of the community, in the form of greater *collaboration and cooperation* between formal and informal mental health practitioners.

### Objective 1: Factors affecting the implementation of CMHFs in the Sierra Leonean context

#### Tradition, beliefs, and culture

Poor knowledge, ‘myths’ and beliefs were repeatedly considered to prohibit mental health programming success. Inferring that these beliefs are ingrained within Sierra Leonean culture and tradition, participants reported that this barrier will require a more long-term approach to overcome:

“I think one of the major reason is because of their knowledge about mental health problem. For us in Sierra Leone there are some beliefs that people have, we say in English ‘myth’. They tell you this is devil and because of devil they thought the hospital cannot do anything, it is no problem with health related issue, people think it’s with tradition so the first people they can contact is the pastor or the traditional healers.” (MHN1)

“Because the misconception, whenever someone has mental illness, you know with these things, the spirits. People believe in that, it is only when psycho-educate them, tell them, it will take time for them to believe you because it is started long time ago, it is not just 1 day you can force them.” (MHN3)

It was evident that alternative options to the traditional system of care were considered a last resort, with participants reporting prolonged attempts to ‘cure’ people with mental health difficulties before referring to a MHN:

“…we recently had a case that we tried for long time to treat with little success. So we transfer the case [to a MHN].” (FGP7 - Traditional healer)

“The traditional healers always keep these people in their own places, for a long time to cure them, whenever they tired or they not able to cure them, they refer to us.” (MHN4)

A loss of earnings for those who have traditionally provided mental healthcare emerged as a barrier to forum attendees engaging with the MHNs and referring patients:

“The traditional people see it as a threat that will lose more patients if they refer to me.” (MHN1)

“As a traditional healer, many such cases are brought to me and in expectation that I would treat them as that used to be my trade. Since I attended the training, however, I no longer treat them and I no longer raise money from that source and it is therefore important that we be encouraged and supported to continue.” (FGP48 - Traditional healer)

#### Health System

Other barriers related to the broader context of the health system, including challenges with service availability, affordability and accessibility:

“We need to talk about availability of services, accessibility of services and affordability of services. So these three is lacking… some do think there is no services for mental health, some do think, yes it’s there but, how can I afford it, how can I access it… So we also need to think about this three things.” (MHN5)

Some of the MHNs stressed the lack of human resources for mental health, how this affects them personally and professionally and acts as a further barrier to mental healthcare:

“It is the mental health nurse singular not plural, we only have one.” (FGP24 - Councillor)

“We are just few in the country in mental health. I alone sometimes can get burdened, even if I decided to go and have a rest I could be there having calls, now because there is awareness, sometimes I feel angered but I say no because before then nobody calls me about mental health. I want calls, but not all of the time.” (MHN5)

Forum attendees also doubted the MHNs’ capacity to provide mental healthcare. This was attributed to the absence of decentralised, local facilities and resources to treat mental health conditions. Participants implied that the non-existence of local facilities and associated poor service quality prohibits the community from engaging with this system of care and acts as a barrier to the success of mental health interventions:

“…the MH nurse but does not have a place to admit or keep mental health patients. How will people be convinced that the MH nurse could deal with the situation.” (FGP3 - Religious leader)

“If there is not a good place, how are the local people supposed to value this program? If the place is good you will not have to persuade people to send their loved ones there. Even recovery will be helped by the existence of a good place for keeping patients.” (FGP25 - Religious leader)

“…there is also the interrelated issue of the effectiveness of the services available in the hospital for treating mental illness and the costs involved in getting the services. When people have a view that the services are not effective they are hardly going to put their money into seeking it.” (FGP42 - Religious leader)

The country’s poor infrastructure, specifically the transportation system and tele-communication channels were also considered a barrier to mental health services:

“…there are times the area where people live is not on a motorway road, most times it’s not… it’s hard for them to get this client to bring them to the hospital, it’s big problem. The travel, those around this vicinity they can easily access. For those up the hills it’s a big challenge.” (MHN2)

“Our terrain is very bad and the current mental health nurse has a big challenge on her hands. She cannot transport patients on a motorbike, she needs a car.” (FGP28 - Mammy Queen)

“Where there is better communication the situation is changing more.” (FGP44 - Youth leader)

All of the MHNs reported that the lack of access they have to medication is a major barrier to service provision. The current system of medication distribution requires many people with mental health difficulties or their families to travel long distances in order to purchase the medication from the government mental health hospital:

“…it’s far from them. It’s in the capitol city (central pharmacy) and somebody living three hundred and something miles from the capitol, who does not have any good income, don’t wish to go down to the Capitol.” (MHN5)

“…because we don’t have medication the relatives came and snatch her away took her to the traditional healer… Unless you call (national person responsible for distribution of psychiatric medication to districts at the time) tell him the case history, then he will maybe send medicine. People when something happens immediately want medicine.” (MHN3)

Poverty and the cost of mental health treatment were considered barriers to service utilisation:

“What prevents people from taking mentally ill persons to see the doctor is sometimes the issue of poverty.” (FGP40 - Religious leader)

“Some of them it is money, if you have the medicines and you ask them to pay, he doesn’t have money, he can’t pay, he needs medicine.” (MHN2)

Political will and government priority towards mental health was considered lacking by many participants, with a reported need for investment both financial and political:

“The mental health situation in the country is not good. It is really not good. The ministries of social welfare and health have not invested enough in it. The problem though is that government has not supported mental health in the country.” (FGP21 – Village Chief)

Interlinked with the perceived lack of political and financial investment MHNs reported having no resources or support to assist them as a significant barrier to providing mental health services:

“…as a professional I am saying I also need to have the support, running costs of offices, the unit, we don’t have, there is nothing. So even if I sit in my office, I see patients coming who want services I can either close the door or go off, cause I just think I have nothing to offer for that person… so many other people have come trying to practice mental health but they lack support so what happened? They went off! It’s not sustainable.” (MHN5)

#### Inclusive approaches

Many of the nurses stressed the importance of participatory approaches to ensure the success of mental health programming. Specifically, including traditional and community leaders was seen as key to addressing some of the aforementioned traditional and cultural challenges:

“…most times it is really better if we go to the community and work with the community leaders, the chiefs, it is better for them to host this workshop in their own community and they invite more people rather than we host this workshop and invite them. When if we have the opportunity to have any support for us to go to this community we host workshop and awareness programme in their community it will be more better, that we hosting it and call them. Involve them. If we left them out it will be really difficult.” (MHN 6)

“When we do it [CMHFs] to the religious leaders, the Imams the pastors and the community leaders, it’s nice, but for example we should come to (local area), the head of the community and others, we get them involved from the beginning, we talk to them, it is much better.” (MHN5)

“Yeah, my opinion what I just really want to recommend is the involvement of them, we should really set this time so that we should be visiting these traditional healers, mammy queens and all these peoples, erm frequently to remind them that we are still here to help, we are still here together with them, we are not trying to take their job from them, more like traditional healers, that we want to work side by side with them, that we want to improve the mental health of the people in the community.” (MHN6)

Moreover, inclusive approaches were seen as particularly useful in order to emphasise the benefits of mental health programmes for betterment of the community. By setting this as the common goal, MHNs felt that they would be seen as less of a threat to traditional healers and other community leaders.

### Objective 2: Perceived changes as a result taking part in the CMHFs

#### Awareness and beliefs

Many participants either reported an increased awareness surrounding mental health or portrayed it in their description of mental health:

“We are increasingly aware now through the trainings (forum)… that mental health is very important, more so than we used to think previously.” (FGP11 - Religious leader)

“Good mental health relates to the health of the mind and body, having good senses and thoughts so that the body is well also. If you have poor mental health you will not be well in the body. The forum showed us that.” (FGP32 - Religious leader)

In contrast some participants, primarily traditional healers and religious leaders, showed a lack of awareness and knowledge of mental health, viewing it as the difference between right and wrong:

“If you do the right thing it is good mental health that makes you do it.” (FGP6 - Religious leader)

“People with mental health problems are unable to do anything right.” (FGP2 - Traditional healer)

While many participants’ aetiological views remain unchanged, some participants did report a change in traditional beliefs and perceptions of mental health conditions:

“It is true that in spite of the rejection of the idea of spirits causing it, they are caused by spirits.” (FGP40 - Religious leader)

“… We believe there are cases that God alone can cure and no amount of modern medicine can cure it.” (FGP10 - Traditional healer)

“…it was place where people were traditionally bound. But after the completion of this training (forum) it has changed, perception of them people has changed even with them the herbalist (a type of traditional healer).” (MHN1)

Some of the forum attendees reported learning about different models of treatment and had an increased awareness of the causes of mental health difficulties aligned to these models:

“We always thought that only herbalist and traditional healers had the means to treat mental illness. It was from the training that we learned that there are people trained… that could treat mental illness.” (FGP25 - Religious leader)

“There are benefits from the training we received. You see, poverty can cause mental health problems… smoking of drugs can cause mental illness.” (FGP33 - Chief)

“One of the things you want to look into with hallucination is that the person is distressed perhaps by a loss of job or a loved one…” (FGP10 - Traditional healer)

Ultimately, the knowledge and information obtained during the CMHFs were subsequently shared by participants in the form of advocacy and community engagement:

“…after the forum I see the people take the information even the churches, the pastors were using it like a sermon, even in the mosque they use it also like sermon.” (MHN1)

“If we have such people in our community, we as leaders have a responsibility to speak out on their behalf. We try now to sensitise the community and the family…” (FGP17 - Religious leader)

“We have added to what they know from our sensitisation activities when we returned home from our training we went and replicated the training in our communities… You can see that there is more understanding on the part of the community people now.” (FGP12 - Traditional healer)

#### Behaviour towards people experiencing psychological distress

Despite moderate changes in participant’s beliefs and awareness of mental health, the continued stigmatisation and exclusion of people with mental health conditions, and negative connotations surrounding mental health difficulties were apparent throughout the data:

“As soon as they see them they refer to them as being lunatics.” (FGP32 - Religious leader)

“To be honest, a mad person has no friend. None of us here would say that when a person with mental health problems that has reached the extreme level would be allowed to be in the same room as him. NO”. (FGP18 - Religious leader) (In response most of the participants of this FGD expressed their agreement)

“None of us would say even if the person were his son, they would like to share the same room with them once they had become completely mental.” (FGP38 - Chief)

Some participants were becoming aware that their actions were stigmatising as a result of participating in the CMHFs:

“…before [the CMHFs] we did not know we were in effect stigmatizing them.” (FGP30 - Traditional healer)

It became apparent that some of the CMHF attendees feared people with mental health difficulties:

“Formerly, I feared them… I still do.” (FGP38 - Chief)

“They said they feared them because they feel that these people are dangerous, they will harm them.” (MHN4)

For many this has changed and the concept of encouragement appeared to replace fear, with some participants even recognising the need for empathy:

“Back then we used to be scared of them. Now we encourage them and speak nicely to them.” (FGP27 - Mammy Queen)

“But now after the training, they said these people are not harmful they are not dangerous they understand they need to show empathy to them”. (MHN2)

Most participants reported that harmful practices and the way people with mental health difficulties are now being treated has changed as a result of the forums. Some traditional practices were often discussed in the past tense:

“Now instead of them being negative, by stoning them, stigmatising them, provoking them, now they are embracing them even when other people want to provoke them they say no, this is against the patient rights.” (MHN2)

“When we came for the workshop, the son of one of my brothers was mentally ill. Each time the illness used to set in we would get him and beat him. But after the workshop (CMHF) I would not let the others or anyone beat him.” (FGP39 - Religious leader)

“Formerly people used to tie them up and beat the mentally ill… But in the training we were taught that medicines are available… So the best we should do is bring them to the health centre instead of beating them or tying them up as if they were bad people.” (FGP23 - Chief)

Changes in traditional treatment methods however, were not corroborated by all participants. The maltreatment and human rights violations of people with mental health difficulties is still occurring as described by a MHN who was called to assist in a local community:

“[I heard] somebody screaming help, help, help in a room. And this guy was mentally disturbed so he was confined. Tied. Chained. A very big chain, that caused physical illness, the limbs got swollen they are sores around the limbs (pointing to ankles). Just like they tie an elephant somewhere. He has been tied in the room for months when I got there.” (MHN5)

“If you are an aggressive person in my community (referring to people with mental health difficulties) and you threaten people we gather the youth and they collectively beat you till you leave the community.” (FGP20 - Youth leader)

Some of the participants, especially the mental health nurses, were acutely aware of the human rights violations that have and are still taking place, and the negative impact this has:

“some of these strategies that they are using now, they are practicing is not humanly accepted… its above human rights, its human rights violation.” (MHN5)

#### Collaboration and cooperation

Participants associated the CMHFs with the development of relationships between forum attendees and the MHNs. All of the MHNs explained how to varying degrees they now work in collaboration with forum attendees and this was corroborated by traditional healers and religious leaders:

“Among the changes is that we have a lot of information sharing amongst one another since the training. The training helped us come closer to one another and to learn.” (FGP2 - Traditional healer)

“Before the forum we did not have good cordiality with the mental health nurse. We did not even know about their existence or the possibility of us working together with them to care for the mentally ill. We were collaborating with fellow traditional healers and we depended almost entirely on herbs which is why many cases took so long with us. But since we came to know them and start the forum (CMHF) the nurse started visiting our communities and she commenced treatment with the cases we had… it has been very different. The forum facilitated that.” (FGP9 - Traditional healer)

“We work in collaboration since we came to know that is her job, when we see cases we know are relevant for her we contact her, there is good collaboration.” (FGP31 - Religious leader)

In addition to closer collaboration between MHNs and the forum attendees the majority of MHNs reported changes to the number of referrals they were receiving as a result of the CMHFs.

“Before the forum (CMHF) I was not getting (referrals)… but after the forum the people take the information… the referral is perfect after the forum.” (MHN1)

“…there has been a lot of change (in reference to referrals) in this time… the chief and the head man in the community is referring to me frequently…” (MHN2)

“Before the training I did not send any cases but since then I send any case that I encounter.” (FGP21 - Traditional birth attendant)

One participant discussed how the increase in referrals offers greater opportunity for individuals, both informal and formal health workers, to work collaboratively:

“Through the referral pathway we can explore a number of options, we can have the traditional option, the spiritual option and also invite the mental health nurse. So we have been using the two prong approach…” (FGP10 - Traditional healer).

## Discussion

**Fig. 1:**
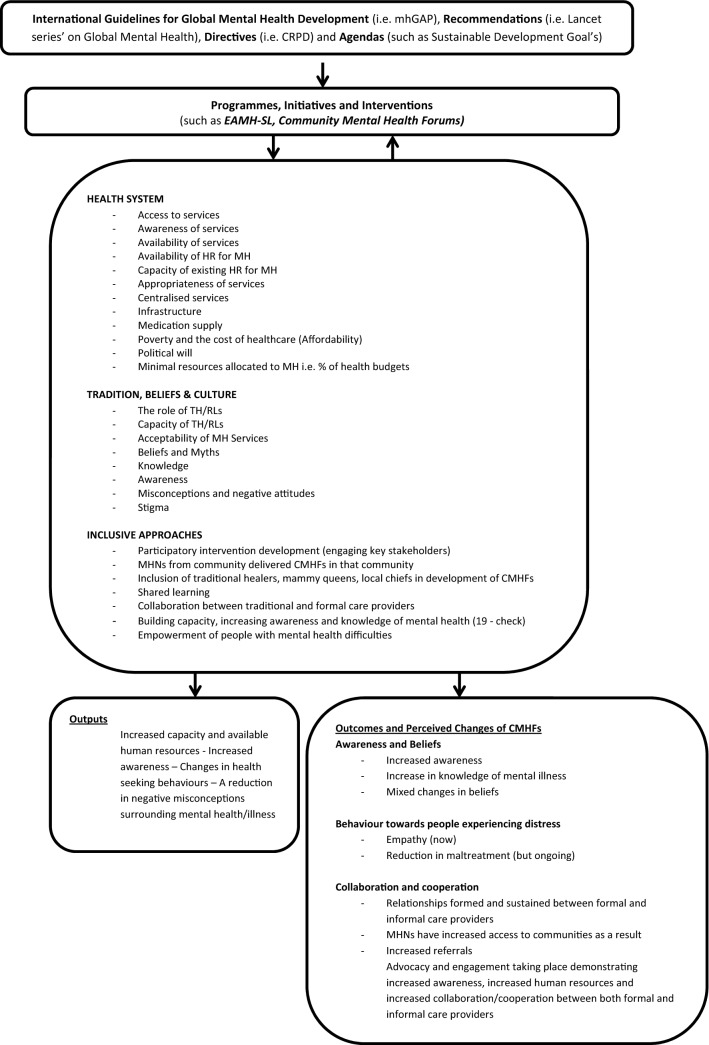
Factors that influence the success of Community Mental Health Forums

Momentum to scale-up access to mental health services and community support structures, reduce mental-health stigma, and address widespread human rights abuses globally is growing [5, 7, 9, 25]. As a result, there has been an increase in the number of community mental health initiatives implemented over the last decade [5]. The CMHF intervention is a form of community engagement, involving both formal and informal mental health care providers, which aims to increase access to quality mental healthcare, empower stakeholders in mental health, and change negative attitudes and behaviours directed at people experiencing mental health difficulties. While participants were more inclined to discuss barriers to the intervention’s success, numerous factors were identified as important for the successful implementation of CMHFs in the Sierra Leonean context. These factors can be considered as falling under one of three themes: *Health System; Inclusive Approaches; and Tradition, Beliefs and Cultural factors*. Figure 1 presents a summary of the factors that influence the success of CMHFs, according to programme participants.

Consistent with systems-thinking and CMH literature [42–44], the factors affecting access to healthcare, in its broader sense, were also found to affect access to mental health care and the successful implementation of community mental health interventions such as the CMHFs. Emerging system-level factors spanned all six of the WHO building blocks [45] including: human resources for mental health (e.g. stigma among MH professionals, burnout among MH professionals, traditional healers as an important human resource for mental health); the availability of medical products and technologies (e.g. medicines); the role of leadership and governance (e.g. poor political buy-in and the importance of community leaders); the lack of communication (e.g. both in terms of information related to mental health, and telecommunications); financing (e.g. poor infrastructure, mental health care as an out-of-pocket health expenditure, loss of income to traditional healers, insufficient government investment); and service delivery (e.g. distances to services, preference for traditional methods, and the inhumane treatment of persons experiencing psychological distress).

In line with previous literature emphasising the important role played by traditional healers and community leaders in alleviating the burden of adverse mental health, participants attributed the accessible, available, and affordable nature of traditional and faith healers as factors contributing to their widespread use and greater patronage [13, 46–48]. While some traditional healers acknowledged the potential benefits of CMHFs, others reported a reluctance to engage with formal care providers due to the loss of earnings associated with collaborating with the MHNs and viewed engaging with formal health systems as potentially undermining to the autonomous nature of their work. The promotion of inclusive approaches was seen by many MHNs as an important mechanism through which to address some of these existing challenges, provide information, dispel myths and stereotypes, and reduce stigma within communities [49–51]. MHNs highlighted the importance of both traditional leaders and formal health workers focusing on a common, agreed upon goal, largely centred around providing interventions, supports and services that benefit the community, which acts as a common motivator across the cadres.

While this was not set up as an evaluative study, several perceived changes were noted as a result of taking part in the CMHFs. Results suggest the intervention had some success, particularly among religious leaders, in challenging harmful beliefs about mental health. Unfortunately, and while changes in mental health awareness were reported by participants, including the recognition of harmful practices, this apparent increase in awareness did not appear to correspond to reductions in maltreatment and stigmatisation of people with mental conditions. Indeed, the majority of forum attendees continued to employ stigmatising terms, and families and communities continued to socially exclude and ostracise those with mental health difficulties. While the mistreatment of those experiencing mental health difficulties is common throughout the world, mistreatment may be further compounded in resource-constrained contexts by out-dated treatment, under-resourced health systems, and poor quality psychiatric services, leading to worse health outcomes. Taken together, the above suggests that there are a number of important challenges, or barriers, to consider in the implementation of mental health programmes in low and middle income countries (LMICs).

Participants also reported the mistreatment of persons with mental health difficulties, including confinement and ‘beatings’, as still taking place in their communities. While the adoption of contemporary (i.e. Western) mental health models does not necessarily lend itself to improved attitudes and treatment of people with mental health difficulties, as evidenced by the significant amount of mental health stigma still prevalent in the Global North [52, 53], improving mental health awareness, or strengthening mental health literacy, is associated with changes in the recognition, management and prevention of psychological distress [54–56]. The belief that mental health conditions have a spiritual aetiology [13], caused by demons or witchcraft, and regarded as a spiritual, supernatural or moral issue [4], may contribute to high levels of stigma and the mistreatment of individuals experiencing psychological distress [4, 7].

Aligned with the aforementioned theme of inclusive approaches, greater collaboration and communication between programme participants emerged as the most consistent change brought on by the use of CMHFs. Specifically, participants perceived CMHFs as providing a space for dialogue and engagement between traditional and formal health practitioners, and in so, resulted in more information sharing, more frequent referrals for services, and ultimately, better working relationships between these groups. It could therefore be argued that the collaborative approach encouraged by the CMHFs fosters a safer and more positive co-utilisation of both formal and informal providers simultaneously, leading to potentially better outcomes for service users [47].

Advocacy and engagement activities also reportedly took place at a local level as a result of the forums. This was corroborated by all participants, with MHNs having observed such activities taking place and forum attendees discussing the sensitisation activities they undertook following the intervention. The forum attendees’ eagerness to vocalise barriers to mental healthcare further evidences increased advocacy for mental healthcare among CMHF participants. Promotion of advocacy and community engagement as a result of the forums is considered a positive outcome, with the potential to facilitate the scale-up of mental health services [30] and challenge system and societal barriers to mental wellness. Specifically, advocacy and community engagement can potentially play an important role in securing additional resources, including more human resources and direct investment in adequate treatment and interventions for people experiencing psychological distress. When individuals and communities engage collectively in thinking, discussing, and helping one another with mental health and social problems, they are more likely to develop collective agency to act on their problems and environment, and develop greater capacity to respond to mental distress within their communities [30]. Furthermore, community engagement and advocacy can enhance social inclusion and reduce stigma by empowering individuals, families and communities [30, 57, 58], although the current study does not offer sufficient evidence that this has taken place at this time, in this context. However, the results do reflect the potential of the CMHF intervention, highlighting the need for more frequent, or refresher CMHFs, as indicated by the participants themselves.

While every effort was made to triangulate findings, the absence of observational data to validate participant’s responses is a methodological limitation of this study [35]. Secondly, there is the possibility that social desirability bias may have influenced some participants’ responses. This is especially the case for the participants who, despite being informed to the contrary, continued to believe the principal investigator was working for CBM International, which was providing financial assistance to programmes involving some of these participants. Third, with no pre-programme (i.e. baseline) data it was difficult to quantify change; thus future evaluations should use a more robust design such as pre-post, or longitudinal design to measure change, including sustainability of change across time. In addition, future research should consider the impact of decentralising CMHFs beyond district level and seek to pinpoint the ideal number of CMHFs, delivered at what frequency, is most effective to promote positive mental health outcomes.

## Conclusion

This study offers a novel framework that considers multiple factors which influence the implementation of CMHFs, in furtherance of the importance of traditional, cultural, and system considerations in the implementation of such programmes. This study further offers insight into the potential of CMHFs to impact on a community’s awareness, beliefs and attitudes towards mental health. Collaboration and cooperation between various stakeholders, whether through the use of CMHFs or other, similar approaches, offer promising opportunities (such as overcoming resource constraints) and means through which to reduce stigma and end the maltreatment of people experiencing mental health difficulties. Therefore, and while the factors that influence the successful implementation of CMHFs are multiple and complex, community-based participatory approaches, whereby community stakeholders are included in all stages of a project’s cycle, are central to the success of community mental health programming [59].

## Supplementary information

**Additional file 1.** Coding summary.

## Data Availability

The datasets used and/or analysed during the current study are available from the corresponding author on reasonable request.

## Abbreviations

CMHF: Community Mental Health Forums
EAMH-SL: Enabling Access to Mental Health Sierra Leone
FGD: Focus group discussion
MH: Mental health
mhGAP: Mental Health Gap Action Programme
MHN: Mental health nurse
MoHS: Ministry of Health and Sanitation
SSI: Semi structured interview
WHO: World Health Organization
CMH: Community Mental Health
