# Supplementary material for: Stakeholder perspectives of Community Mental Health Forums: a qualitative study in Sierra Leone
Source: Int J Ment Health Syst. 2020 Jul 10;14:50. doi: 10.1186/s13033-020-00382-7 (PMC7350667; doi:10.1186/s13033-020-00382-7)
Supplement: Supplementary file 1 — Additional file 1. Coding summary. [file 13033_2020_382_MOESM1_ESM.docx]

*Additional file 1. Coding summary.*

| **CODING 1**  (as identified in the literature - theoretical framework) | **CODING 2**  (emerging codes) | **CODING 3 & 4**  (axial codes/categories & selective examination) |
| --- | --- | --- |
| **Objective 1: Factors affecting the successful implementation of CMHFs in the Sierra Leonean context** | | |
| Barriers:   - Limited access to services - Resources - Lack of knowledge - Limited awareness - Stigma - Pluralistic systems of care - Cultural beliefs   Facilitators:   - Participatory approach/stakeholder involvement - Community engagement - Changes in awareness and knowledge - Collaboration - Advocacy activities | Economic barriers:   - Service affordability - Associated loss of earnings for TH/RL - TH/RL considered cheaper than formal care - Transport to services cost - Communication costs   Infrastructure barriers:   - Travel to services (distance and means) - Communication means (access to mobile phones) - Closer proximity of TH/RL compared to formal services   Traditional/Cultural barriers:   - TH/RL feared MHNs wanted to “take their knowledge” - THs/RLs as gatekeepers to communities and individuals - Formal care is last resort - Stigma associated with being a MH professional and self-stigma among MH workers   System Barriers:   - Poor condition of and treatment by formal health services prevents people engaging with them i.e. chaining to beds (linked to service quality) - Lack of trust/faith in MHNs as a result of under resourced services with poor outcomes - Health staff (inc. MHNs) at times fear for their safety and feel at risk when with MH service users - Frustration and burn-out of formal care staff   Facilitators:   - Participatory intervention development - MHNs from community delivered CMHFs in that community | **HEALTH SYSTEM**   - Access to services - Awareness of services - Availability of services - Availability of HR for MH - Capacity of existing HR for MH - Centralised services - Infrastructure - Medication supply - Poverty and the cost of healthcare (Affordability) - Political will   **TRADITION, BELIEFS & CULTURE**   - The role of TH/RLs - Acceptability of MH Services - Beliefs and Myths - Knowledge - Awareness - Misconceptions and negative attitudes - Stigma   **INCLUSIVE APPROACHES**   - Participatory intervention development - MHNs from community delivered CMHFs in that community - Inclusion of traditional healers, mammy queens, local chiefs in development of CMHFs |
| **Objective 2: Perceived changes as a result of taking part in the CMHFs.** | | |
| Outputs:   - Increased capacity, knowledge and awareness - Changes in health seeking behaviours - Changes in perceptions/beliefs surrounding mental health/illness   Outcomes:   - Increased access to formal MH care - Increase referrals and patient numbers - A reduction in stigma and the mistreatment of people with MH difficulties | Outputs:   - Promoted self-awareness and reflexivity among attendees of CMHFs - Willingness to accept other treatment modalities - Less likely to accept alternative explanations surrounding cause of mental health conditions - Advocacy and community engagement activities being carried out by CMHF attendees as a result of intervention   Outcomes:   - Minimal reduction in stigma as a result of CMHFs (in line with literature and findings from other one-off interventions) - Reported significant reduction in maltreatment of people with mental difficulties by informal care providers - Increase in referrals and caseloads demonstrating increased access and service utilisation | **Awareness and Beliefs**   - Increased awareness - Increase in knowledge of mental health - Mixed changes in beliefs   **Behaviour towards people experiencing distress**   - Stigma ongoing - Fear (previously) - Empathy (now) - Reduction in maltreatment (but ongoing)   **Collaboration and cooperation**   - Relationships formed and sustained between formal and informal care providers - MHNs have increased access to communities as a result - Increased referrals - Advocacy and engagement taking place as a result of the forums demonstrating increased awareness, increased human resources and increased collaboration/cooperation between both formal and informal care providers |
